# Multidisciplinary Management of Sternal Osteomyelitis Due to *Klebsiella aerogenes* after Open Heart Surgery in a Patient with Multiple Myeloma: A Case Report and Discussion of the Literature

**DOI:** 10.3390/microorganisms11112699

**Published:** 2023-11-03

**Authors:** Marco Pignatti, Giampiero Dolci, Elena Zamagni, Renato Pascale, Ottavio Piccin, Alessandro Ammar, Flavia Zeneli, Maria Elisa Lozano Miralles, Katia Mancuso, Riccardo Cipriani, Pierluigi Viale, Davide Pacini, Sofia Martin-Suàrez

**Affiliations:** 1 Plastic Surgery, IRCCS Azienda Ospedaliero-Universitaria di Bologna, 40138 Bologna, Italy; alessandro.ammar@icloud.com (A.A.); flaviazeneli@yahoo.com (F.Z.); mariaelisa.lozano@gmail.com (M.E.L.M.); riccardo.cipriani@aosp.bo.it (R.C.); 2 Department of Medical and Surgical Sciences (DIMEC), University of Bologna, 40138 Bologna, Italy; e.zamagni@unibo.it (E.Z.); renato.pascale2@unibo.it (R.P.); katia.mancuso3@unibo.it (K.M.); pierluigi.viale@unibo.it (P.V.); davide.pacini@unibo.it (D.P.); 3 Thoracic Surgery, IRCCS Azienda Ospedaliero-Universitaria di Bologna, 40138 Bologna, Italy; dolci.giampiero@gmail.com; 4 Haematology, IRCCS Azienda Ospedaliero-Universitaria di Bologna, 40138 Bologna, Italy; 5 Infectious Disease Unit, IRCCS Azienda Ospedaliero-Universitaria di Bologna, 40138 Bologna, Italy; 6 Otorinolaryngology, IRCCS Azienda Ospedaliero-Universitaria di Bologna, 40138 Bologna, Italy; ottavio.piccin@unitn.it; 7 Plastic Surgery, Policlinico di Modena, University of Modena and Reggio Emilia, 41126 Modena, Italy; 8 Cardiac Surgery, IRCCS Azienda Ospedaliero-Universitaria di Bologna, 40138 Bologna, Italy; sofia.martinsuarez@aosp.bo.it

**Keywords:** sternal osteomyelitis, open heart surgery, multiple myeloma, microsurgical flap, chest reconstruction

## Abstract

Sternal wound complications following cardiac surgery, including sternal dehiscence, mediastinitis, and osteomyelitis, pose significant challenges in terms of management and patient outcomes. We present a case report highlighting the complex management of a patient who underwent open heart surgery for severe aortic valve stenosis, followed by sternal wound dehiscence and sternum osteomyelitis due to extended spectrum beta lactamase (ESBL) producing *Klebsiella aerogenes*. A multiple myeloma diagnosis was also suspected at the positron emission tomography (PET) scan and confirmed with bone marrow biopsy. Multidisciplinary evaluation of the case led to a comprehensive treatment plan. To control the sternal osteomyelitis, total sternectomy was performed followed by immediate reconstruction with a bone (tibia) graft from the tissue bank and fixation with the minimal hardware possible. A microsurgical latissimus dorsi free flap was required to reconstruct the soft tissue defect. After 6 weeks of antibiotic treatment with ertapenem and fosfomycin based on a culture of intraoperative material, no clinical, imaging, or laboratory signs of infection were seen. Multiple myeloma treatment was then started. At 1 year of follow up, no recurrence of infection occurred, and the reconstruction was stable and closed. Multiple myeloma is under chronic treatment with novel agent combination, with an excellent haematological response.

## 1. Introduction

Sternal wound infection (SWI) is a serious complication of cardiac surgery, causing a delayed healing of wounds and evolution towards life-threatening complications such as sternal osteomyelitis (SO) and mediastinitis [1,2]. SWI and SO incidence varies between 0.5% and 8% of patients undergoing cardiac surgery [1]. 

Sternal wound infections are classified according to the anatomic location of the infected tissue. Specifically, deep SWI are categorized into several types: infections extended below the fascia without involvement of the bone or retrosternal tissue; infections involving retrosternal tissue, bone, and retrosternal tissue; or causing frank SO [3]. SO is the rarest form of deep SWIs and the more complex type in regard to the diagnosis, management, and outcome. Unlike SWI, which often presents with frank wound dehiscence, SO is more subtle in its clinical presentation. SO may present as purulent draining sinus tracts without any clinical signs and as a closed sternal wound. Fistulas often occur several weeks or even months after surgery and apparently resolve after a short course of antibiotic treatment, resulting in recurrence [1,4].

Sternal wound complications occur in 0.8% to 8% of patients; however, the exact incidence and risk factors for SO have not been studied yet [1]. Probably, the misleading diagnosis could cause difficulties in determining the true incidence of SO.

Gram-positive bacteria (GPB) are the main causative agents, accounting for 80% of cases of SWI and SO [5,6]. However, Gram-negative bacteria (GNB) are emerging as frequent causative pathogens of these challenging infections, often with limited therapeutic options due to difficult susceptibility antibiotic profiles [7,8].

The cardiosurgical procedures have been improving over the years through the implementation of new technologies and safety standards [9,10]. As a consequence, patients undergoing cardiac surgery procedures often have complex comorbidities, posing them at potentially higher risk of infection complications.

We present a case report highlighting the complex management of a patient with underlying plasma cell dyscrasia present from several years who underwent open heart surgery for severe aortic valve stenosis, followed by SWI and SO.

Moreover, we describe the challenges associated with the management of these infections, complicated with a concomitant diagnosis of multiple myeloma.

The literature does not offer univocal detailed recommendations on the treatment of SO although thorough debridement and antibiotic treatment specific to the cultured microorganism seem to be the mainstay, even if literature data on antibiotic choice and duration of therapy are scarce and management should be expert-driven.

Sternal and soft tissue reconstruction can also be achieved with multiple methods, depending on site, extension of the defect, patient’s comorbidities, surgeon experience, and hospital setting.

A timely diagnosis, interdisciplinary collaboration, and tailored treatment strategies are of outmost importance to optimize patient outcomes especially when concomitant diseases complicate the treatment.

## 2. Case Report

A 49-year-old gentleman was admitted to our Cardiac Surgery Unit for severe aortic valve stenosis. The patient’s medical history included kappa light chain monoclonal gammopathy of undetermined significance (MGUS), recently evolved in smouldering multiple myeloma (SMM) and glaucoma. No cardiovascular risk factors were reported.

In August 2021, the patient underwent aortic valve replacement surgery with a median sternotomy approach. 

Eleven days after surgery, the patient developed a fever. Blood cultures revealed extended spectrum beta lactamase (ESBL) *Klebsiella aerogenes* infection. Prolonged infusion of meropenem was started according to an antimicrobial susceptibility test (meropenem MIC ≤ 2 mg/L, ertapenem MIC ≤ 0.5 mg/L).

**Figure 1:**
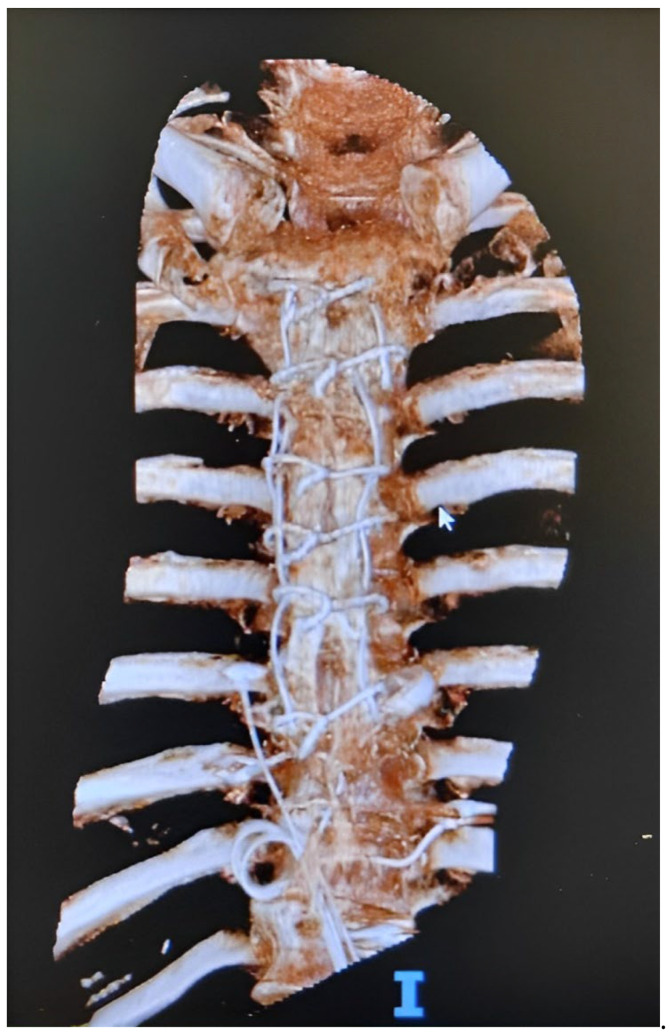
Preoperative 3D CT scan—outcomes of sternotomy and sternal wiring from the previous surgery are visible.

A few days later, signs of sternal wound dehiscence appeared. After a computer tomography (CT) scan excluding bone involvement, the patient underwent a surgical debridement and wound closure. Sternal synthesis materials were not removed during surgery due to the sternal instability and absence of SO at imaging (Figure 1). Intraoperative samples were all positive for ESBL producing *Klebsiella aerogenes* and meropem treatment was continued.

In addition, negative pressure therapy (NPT) was applied before wound closure. After 2 weeks of treatment, antibiotic therapy was stopped, and the patient was discharged with indication for outpatient dressings due to residual minimal sternal dehiscence. 

Unfortunately, in October 2021, the patient was readmitted to the Cardiac Surgery Unit due to the appearance of sternal fistulae. Due to the high suspicion of SO, a CT scan was performed, confirming a little area of sternal bone involvement. Therefore, a sternal bone surgical debridement was performed. Subsequently, NPT was applied. Bone intraoperative samples again revealed *Klebsiella aerogenes* growth with the same antimicrobial susceptibility profile. Ertapenem therapy was started. After 6 weeks of antibiotic therapy, an improvement in the local condition was observed so ertapenem therapy was concluded and the patient was discharged.

**Figure 2:**
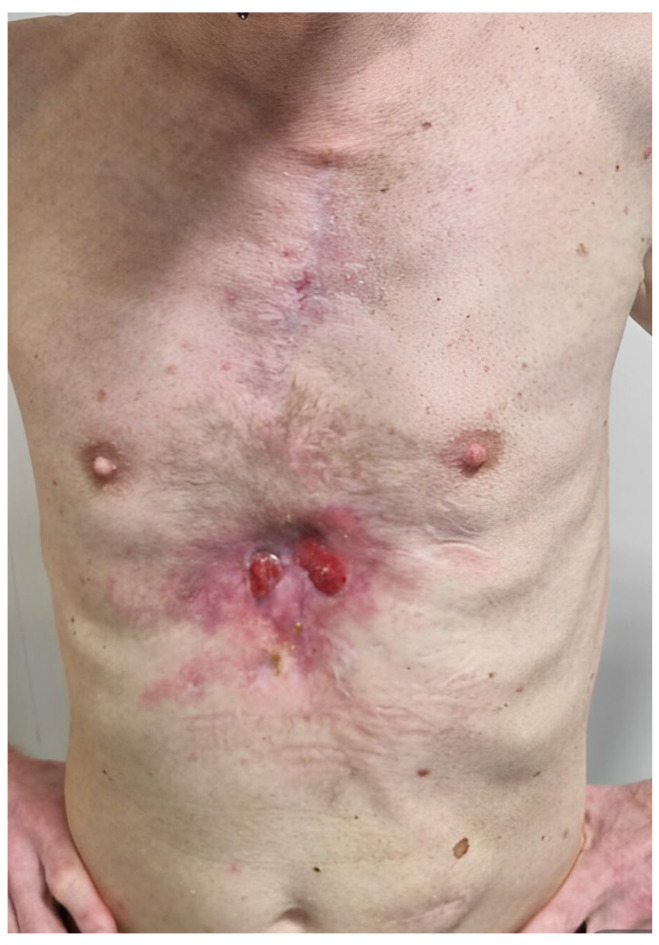
Sternal wound dehiscence.

**Figure 3:**
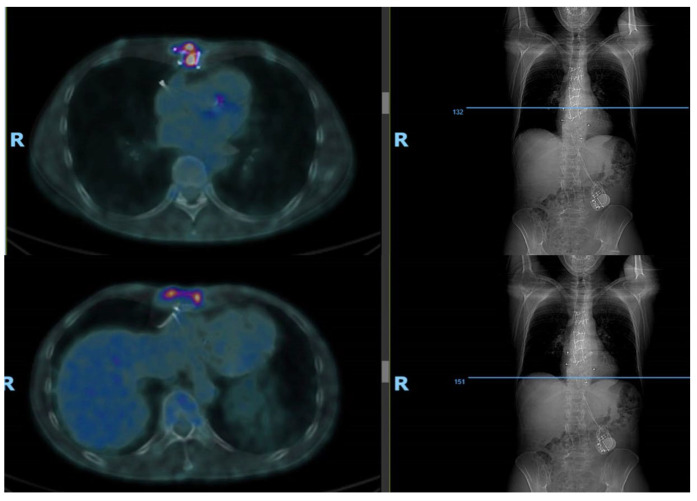
Osteomyelitis diagnosis—CT PET images documenting sternal osteomyelitis at various sections of sternum (proximally and distally).

**Figure 4:**
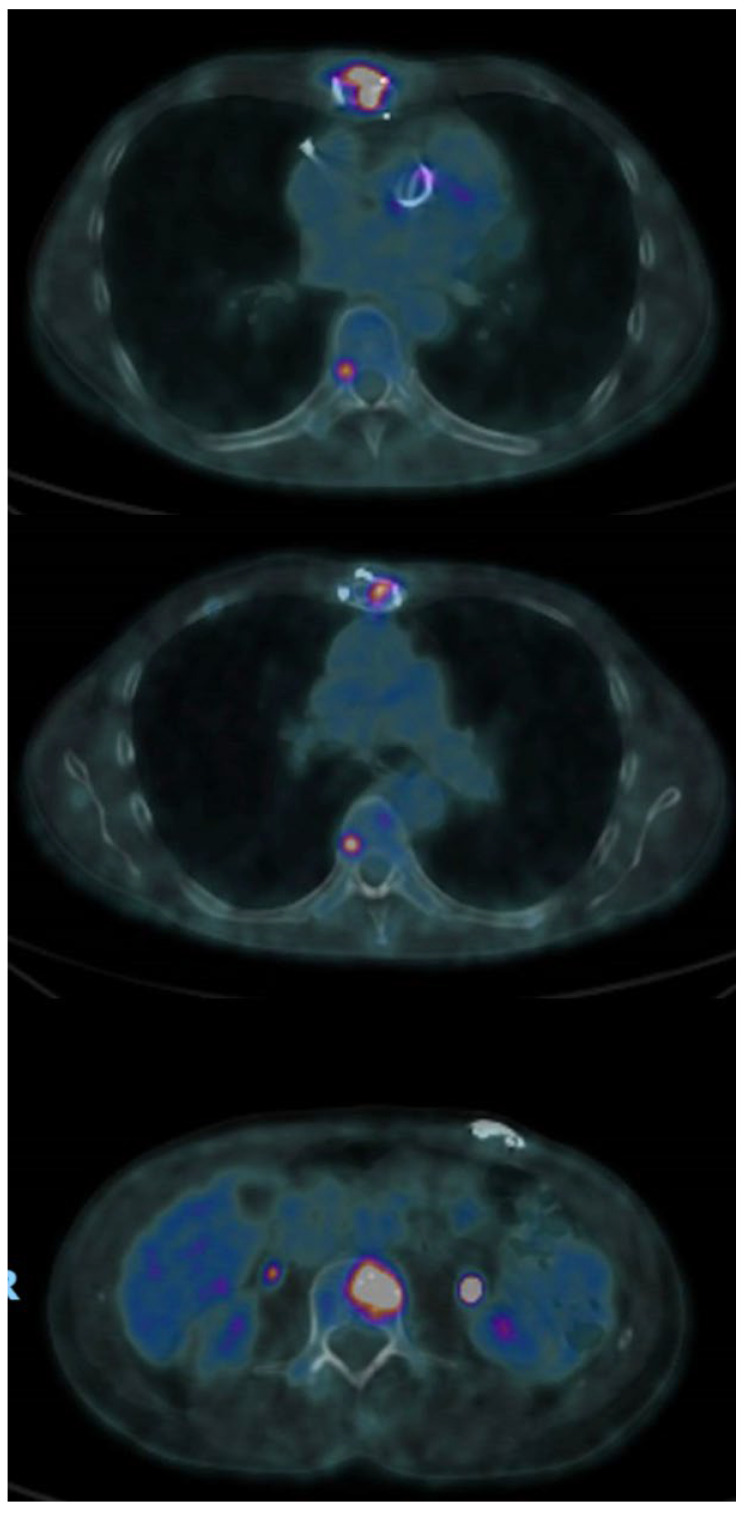
Multiple myeloma—CT PET image shows multiple focal areas of vertebral bone marrow with increased metabolic activity at T6 (**above**), T10 (**middle**), L1 (**below**).

In April 2022, the sternal wound dehiscence with fistulae relapsed (Figure 2). A microbiological culture swab was repeated and confirmed a recurrence of ESBL *Klebsiella aerogenes* infection. Fluorodeoxyglucose positron emission computed tomography (FDG PET/CT), already planned for the follow up of the SMM, was also used to investigate the sternal bone status. Radiological findings confirmed the diagnosis of SO relapse involving the entire bone from manubrium to xifoid (Figure 3). Additionally, FDG PET/CT showed multiple areas of bone captation suspicious for the progression of smouldering to active multiple myeloma (MM) (Figure 4). MM was then confirmed with a complete staging of the disease, including bone marrow biopsy conducted by haematologists.

The case was discussed by the multidisciplinary team (MDT) composed of Cardiac, Plastic, and Thoracic Surgeons; Infectious Disease Specialists; and Haematologists. The need of surgical debridement of the infected bone faced the challenge of choosing the adequate treatment plan for the osteomyelitis and for MM. 

According to age and fitness, the treatment of haematologic neoplasia would include quadruplet-drug combination as induction, consisting in anti-CD38 monoclonal antibody daratumumab, bortezomib, thalidomide, and high-dose dexamethasone (Dara-VTd), followed by stem cell mobilization and collection, and high-dose chemotherapy with melphalan, in preparation for autologous stem cell transplantation (ASCT). 

The MM treatment was considered at high risk due to the concomitant active infection of the sternal bone. Addressing the infection right away would have required a complex operation and a lengthy recovery that would have postponed the treatment of MM.

The decision was taken to obtain as a first aim the treatment of the infection and wound healing, before starting haematological therapy, relying also on the low risk of disease progression in a few months for standard-risk MM.

Cardiac and Thoracic Surgeons performed an extensive debridement with total sternectomy followed by empirical ertapenem and intravenous fosfomycin treatment (fosfomycin MIC ≤ 32 mg/L, agar dilution method on previous isolates). Antibiotic treatment was confirmed after the growth from the bone intraoperative sample of the well-known *Klebsiella aerogenes* strain. 

**Figure 5:**
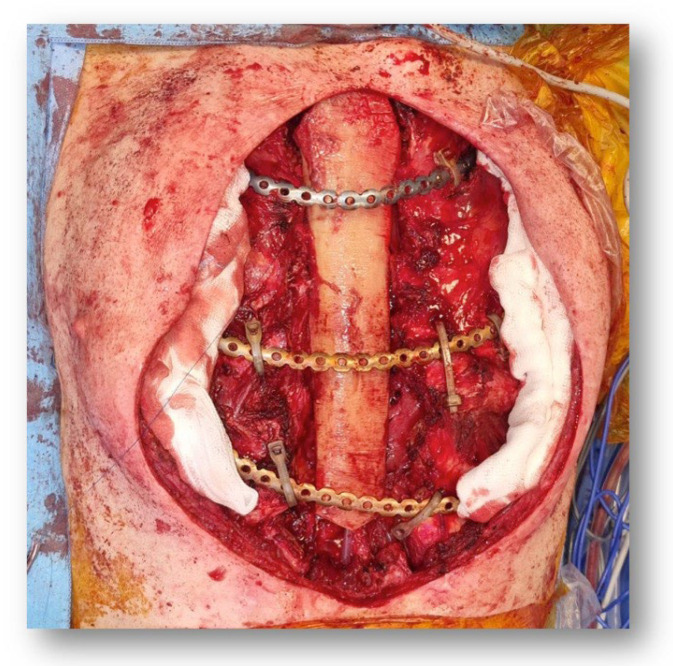
Sternum reconstruction—intraoperative photo of the bone allograft used to reconstruct the sternum and fixed to the costal stumps with titanium plates.

To reconstruct the sternum, a bone allograft was implanted and fixed with rigid titanium costal plates (Figure 5). Sternoclavicular joints were reconstructed using Sternal ZIPFIX^TM^ System (DePuy Synthes).

The wide soft tissue defect was repaired by Plastic Surgeons using a free latissimus dorsi (LD) flap, which was anastomosed to the internal mammary vessels.

Unfortunately, the flap suffered venous congestion overnight, leading to the need for a reoperation. 

**Figure 6:**
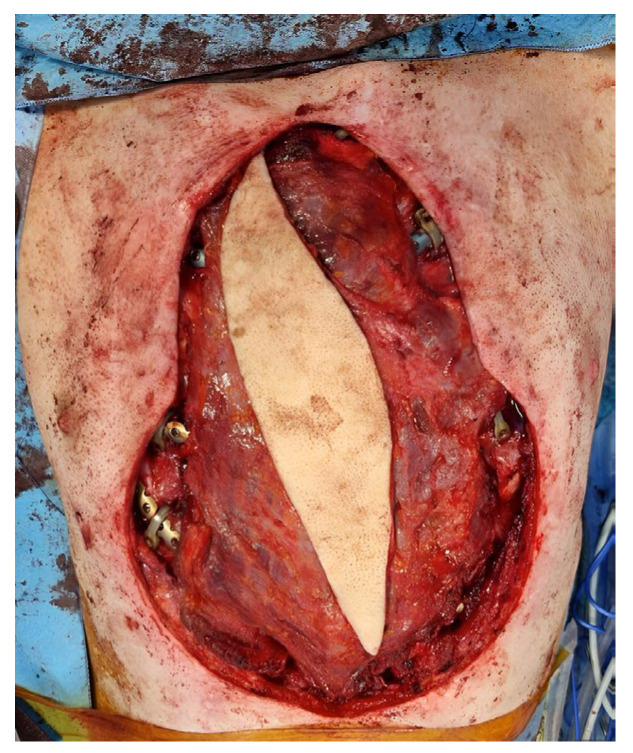
Latissimus dorsi flap—intraoperative photo of the LD flap to cover the allogenic bone graft and costal plates. A skin paddle of size 20 cm × 8 cm was harvested and the lateral areas were then grafted with meshed split-thickness skin grafts from the patient’s thigh.

The following day, the necrotic LD flap was removed, and the soft tissue defect was repaired with the contralateral LD free flap (Figure 6). The flap was in-set with the pedicle positioned cranially, allowing the artery and vein to be anastomosed to the transverse cervical artery and external jugular vein, through a subcutaneous tunnel to reach the neck.

No complication occurred. After 14 days, due to the good healing of the flap and thoracic wounds, the patient was transferred to the Infectious Disease Unit to continue antibiotic therapy. Subsequently, the patient was discharged (3 weeks after the surgical procedures). Fosfomycin was stopped and monotherapy with ertapenem was continued for another 3 weeks at the Infectious Disease Outpatient Service. After a total of 6 weeks, the antibiotic treatment was stopped. 

**Figure 7:**
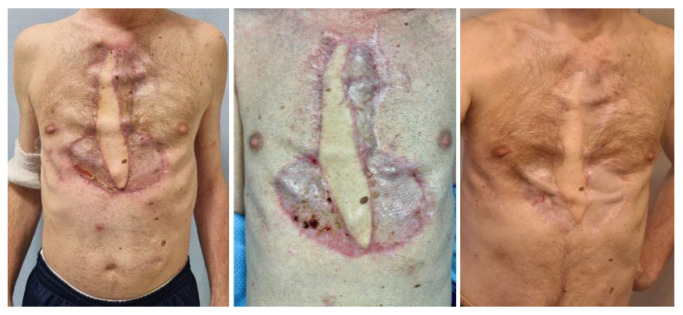
Postoperative follow up—(**left**) at 3 months, wounds are healed. (**center**) At 6 months, the flap is stable. (**right**) At 12 months, LD muscle hypotrophism is observed, but no hardware exposure or infection relapse occurred.

At 30 days from surgery, the MM treatment was simultaneously started. The patient received the four planned induction cycles, obtaining a partial response. Unfortunately, he failed to collect enough haemopoietic stem cells to perform ASCT; the insufficient stem-cell availability might be due to the prolonged antibiotic therapy, together with the removal of the sternum, which is one of the sites with more active haematopoiesis/red bone marrow. Therefore, the patient resumed a quadruplet therapy for an additional two cycles, substituting thalidomide with lenalidomide, attaining a very good partial response, then going on with continuous treatment with daratumumab–lenalidomide–dexamethasone, according to current guidelines for non-transplant-eligible patients with MM [11]. At 1 year from treatment initiation, the patient was in good clinical conditions (Figure 7), with an optimal drug tolerance and in complete remission.

## 3. Discussion

Sternal wound complications following cardiac surgery pose significant challenges in terms of management and patient outcomes. In this case, the patient’s underlying MM added further complexity to the treatment plan.

Risk factors associated with deep sternal wound infection (SWI) are older age, obesity, smoking, diabetes, chronic lung disease, concomitant coronary artery bypass grafting with a valve or aortic surgery, long operation time, the bilateral use of internal mammary arteries, and postoperative prolonged ventilator and inotropic support [12,13,14].

In our case, possibly the underlying haematological condition, a disease involving B lymphocytes and plasma cells, inducing B-cell function impairment and immune paresis, contributed right from the first heart surgery to the impaired wound healing and increased susceptibility to infections, especially the SO caused by ESBL-producing *Klebsiella aerogenes*.

Regarding aetiology, GPB (*Staphylococcus aureus* and Coagulase-negative *Staphylococcus* spp.) are the most frequent etiological strains responsible for deep SWI [5,6]. GNB are less frequently responsible for SWI, approximately one in four cases compared with GPB, and are frequently polymicrobial (20–44%) [15]. The most common GNB involved are Enterobacterales (*Escherichia coli*, *Klebsiella* spp., *Serratia* spp., *Proteus* spp., *Citrobacter* spp.), but increasing episodes caused by *Acinetobacter* spp., *Pseudomonas aeruginosa*, and *Stenotrophomonas maltophilia* have also been described [16]. SO due to GNB seem to be associated with a high rate of complications such as drainage failure, prolonged mechanical ventilation, and an increase in the mortality rate [7,17]. The worst outcome might be due to an inappropriate initial antimicrobial therapy [8]. In our case, appropriate antibiotic therapy against *Klebsiella aerogens* was promptly started empirically after each surgical revision, on the base of previous cultures, while waiting for new results.

In regard to pathogenesis of SO, contamination of the wound from the margins is the first cause of SO due to GPB, common components of the skin flora (and hospital environment) [18]. Conversely, the mechanism of wound inoculation with GNB is unclear. GNB aetiology has been shown to be associated with the same risk factors of GPB such as prolonged mechanical ventilation or a complicated postoperative course. In addition, some authors suggest as risk factors for GNB infection the use of a vein graft harvested from a contaminated donor site in coronary artery bypass surgery [19] or intraoperative supplies contamination. Recently, an outbreak of deep SWI caused by Serratia marcescens due to the use of a contaminated aqueous chlorhexidine solution has been reported [20]. However, these last hypotheses are not applicable to our case. Indeed, GNB are often responsible for another concomitant focus of infections, especially pneumonia, urinary infection, or bacteraemia that could be the cause of subsequent SO development [19,21]. An early postoperative bloodstream infection seems more likely in our case. 

*Klebsiella aerogenes*, formerly known as *Enterobacter aerogenes* [9], is responsible for a broad range of clinical syndromes, such as bacteraemia, skin and soft tissue infections, and respiratory tract, urinary tract, and gastrointestinal tract infections. Among these clinical syndromes, osteomyelitis is a relatively rare type of infection [8].

In regard to diagnostic investigations, a CT scan is the most frequent tool used for SO by visualizing the pathological bone alterations such as cortical destruction, heterogeneous bone density, and bony sequestra [22]. FDG PET/CT is a diagnostic tool in several infectious diseases such as prosthetic joint infections, vertebral osteomyelitis, or vascular prosthesis infection and cardiac implantable electronic devices (CIED) infection [23,24]. The role of FDG PET/CT also seems to be promising for SO diagnoses [25]. Furthermore, excision of all infected and necrotic bone is crucial to obtain resolution of SO. It is well known that FDG PET-CT could be used as a precise and accurate tool to localize infected tissue and consequently to plan the proper debridement plan [26]. In addition, in several reports, the yield of FDG PET/CT seems to be higher when infection is caused by GNB than when it is caused by GPB [27,28]. In our report, FDG PET/CT was used not for the diagnosis of SO but for the follow up of the haematological disease. The finding of pathological uptake at the sternal level confirmed the role of this tool for the SO diagnosis. 

Concerning the surgical management of sternal dehiscence, the algorithm of Cauley et al. [29] suggests a stepwise evaluation and intervention plan, with debridement, negative pressure wound therapy, and reconstructive procedures when indicated. This approach was followed in our case with multiple revision surgeries, including debridement and negative pressure therapy, before considering a more aggressive surgical strategy. 

The treatment was finally successful thanks to a radical debridement with the removal of all nonviable tissue and all hardware as usually recommended for the surgical management of osteomyelitis [30,31]. 

According to a recent review of Hamaguchi et al. [31], early flap reconstruction has shown improved survival outcomes compared to cases treated with sternal rewiring alone. 

As is usual, the main limitation to an aggressive surgical treatment of a sternal wound dehiscence lies in the often mild clinical presentation and modest soft tissue defects presented by these patients, who frequently have compromised general health conditions.

However, despite these sensible reasons for a less aggressive surgical approach, a generous early debridement, removal of all synthetic material, and a prompt involvement of the Thoracic and Plastic Surgeon may lead to a successful treatment of wound dehiscence, sternal osteomyelitis, and mediastinitis.

After surgical debridement, the other main measure to be used for the treatment of SO is antibiotic therapy based on intraoperative specimens. In case of infection with ESBL-producing Enterobacteria, the treatment with carbapenem is recommend with international guidelines [32,33]. Among carbapenems, ertapenem is an acceptable option and can be useful in the outpatient setting thanks to the once-daily dosing [10]. In our case, it was chosen as the backbone for the treatment of SO. Additionally, we administered intravenous fosfomycin for 3 weeks as combination therapy. 

Combination antibiotic therapy is not routinely recommended for infections caused by ESBL Enterobacterales [32,33]. However, in our case, the use of empiric combination therapy was justified due to the high risk of mutant selection after prolonged exposure to carbapenems with inadequate source control. After the culture results, the use of fosfomycin as a second agent was continued for another 2 weeks due to the PK/PD characteristics of this drug. Indeed, intravenous fosfomycin has high activity against GPB but also against ESBL-producing *Enterobacterales*, particularly in combination with other antibiotics [34]. Furthermore, fosfomycin has high antimicrobial activity under low-oxygen conditions and low pH and a sufficient penetration into abscess fluid, characteristics of an infected bone environment. In addition, fosfomycin seems to achieve high bone concentrations also due to its chemical structure similarity with hydroxylapatite, promoting distribution into the inorganic part of bone [35,36,37,38].

However, for the treatment of SO, especially in the setting of GNB, the literature does not provide definitive recommendations on which antibiotic should be chosen and for how long it should be continued [39,40]. 

The duration of SO therapy generally ranges from 4 to 6 weeks and depends on the extent of surgical debridement performed. Conversely, if sternal debridement has not been performed, up to 12 weeks of antimicrobial therapy are described [41]. However, clinical evaluation, evolution of inflammatory markers, microbiological tests, and imaging studies should guide clinicians rather than fixed terms, especially if the need for a new surgical approach seems necessary. The choice between intravenous or oral-route drugs should be taken according to the susceptibility testing, oral bioavailability, and tolerance [42]. In the absence of available oral-route drugs, intravenous treatment could be maintained as outpatient antimicrobial treatment. In our experience, after an extensive surgical debridement with removal of the infected sternal bone, a 6-week course of antibiotic therapy is usually effective.

Reconstruction of the anterior chest wall after total sternectomy and extensive tissue removal in patients with osteomyelitis may be challenging as it aims to provide skin closure, foreign material coverage, and organ protection while allowing effective respiratory movements. 

Different techniques and materials have been described. However, given the complexity of the issue, none of them are yet considered the gold-standard procedure [43,44].

Some authors have proposed reconstruction of the anterior chest wall with musculocutaneous flaps alone (pectoralis major, latissimus dorsi, rectus abdominis) without bone replacement. This technique avoids prosthetic materials and supplies highly vascularized muscle tissue, allowing optimal infection control. However, the lack of an underlying solid structure may cause inadequate protection of the underling organs and insufficient support for respiratory dynamics with possible paradoxical movements of the chest wall [44,45,46].

Rigid reconstruction with different materials (methacrylate, silicone, cyanoacrylate mesh, and titanium plates) has widely been used, but the main disadvantages may be a limitation of respiratory movements and adjacent tissue erosion [47,48]. 

Recently, a few cases of 3D implants have been reported with apparently good results. Although the custom-made shape reduces the risk of rigidity and erosion, the limited follow-up data and the high production cost have to be considered [49]. In addition, the presence of prosthetic materials increases susceptibility to infectious complications especially in patients with osteomyelitis [50].

In our case, we preferred to provide a skeletal reconstruction by using a cryopreserved allogenic tibial bone graft fixed with rigid titanium costal plates. 

The previous unsuccessful attempts of partial debridement and the urgent need to start multiple myeloma treatment led to choosing a treatment plan that could provide, despite the surgical risks and technical difficulties, infection eradication and a stable reconstruction with rapid healing. Compared to synthetic materials, bone grafts (autograft and allograft) seem to have a lower risk of infection and rejection as they serve as a scaffold for osteoprogenitor cells and they eventually integrate into the host tissue [51]. While autologous bone grafts (fibula, ribs, iliac bone) are useful in small defect reconstruction, allogenic grafts provide a greater stock of bone for extensive defects without donor site morbidity. Several studies have reported total sternal allograft reconstruction with optimal results in terms of infection control, physiologic chest function, and long-term graft survival [44,51,52,53]. In addition, in this particular case, bone metabolism was impaired by the underlying haematological disease, already showing signs of increased osteoclastic activity and reduced osteoblast function, thus making an autologous graft less appealing.

The treatment of haematologic neoplasia was another important challenge in this complex clinical case, both for the timing and the choice of therapy. While the up-front treatment of MM is currently still tailored according to age, with patients below 70 years old referred for ASCT, the availability of newer triplets or quadruplets of novel agents and immune therapy is nowadays challenging the need for a transplant, considering the excellent results obtained with a continuous therapy. Therefore, in our patient, the inability to collect peripheral stem cells, due to the detrimental impact of prolonged antibiotic therapy on haematopoietic progenitors, and to the removal of part of the active haematopoietic tissue with the sternum removal, did not impair a successful outcome of MM. 

## 4. Conclusions

In conclusion, our report highlights the need for a multidisciplinary approach to treat complex infections such as SO, especially in frail patients. Furthermore, we emphasize the need for aggressive surgical treatment followed by tailored antibiotic treatment to have a better chance of eradicating OS.

## Data Availability

The clinical data of the patient are not available for privacy reasons.
